# Relapse prediction and individualized treatment-effect modeling in relapsing multiple sclerosis: a systematic review

**DOI:** 10.3389/fpubh.2026.1940704

**Published:** 2026-09-04

**Authors:** Martin Budil, Martin Rožánek, Petra Petrová

**Affiliations:** Department of Biomedical Technology, Faculty of Biomedical Engineering, Czech Technical University in Prague, Kladno, Czechia

**Keywords:** machine learning, multiple sclerosis, precision medicine, prognostic models, relapse prediction, treatment-effect heterogeneity

## Abstract

**Introduction:**

Individualized prediction of relapse-related outcomes may support treatment selection and monitoring in relapsing multiple sclerosis. However, existing studies differ substantially in clinical purpose, target outcomes, modeling approaches, and validation strategies.

**Methods:**

We conducted a systematic review in accordance with PRISMA 2020. PubMed, Web of Science, IEEE Xplore, and Scopus were searched for English-language records published from 1 January 2010 to 14 January 2026. Studies developing or evaluating models for relapse or relapse-related outcomes in relapsing multiple sclerosis were included. Data on study design, predictors, modeling approach, target outcome, calibration, and validation were extracted. Owing to clinical and methodological heterogeneity, a structured narrative synthesis was performed. The protocol was registered in PROSPERO (CRD42024625392).

**Results:**

Fourteen studies were included: five conventional relapse-prognosis studies, four individualized treatment-effect prediction studies, and five exploratory relapse-related studies. Clinically interpretable models based on structured clinical data generally demonstrated moderate discrimination but more transparent validation. Studies reporting very high predictive performance were commonly based on smaller samples, high-dimensional data, or limited independent validation. Calibration and external validation were inconsistently reported.

**Discussion:**

Relapse-related prediction in multiple sclerosis is feasible, but current evidence remains heterogeneous and insufficiently validated for routine clinical implementation. Progress will require harmonized outcome definitions, consistent calibration reporting, transparent model evaluation, and external validation across clinically diverse populations.

## 1 Introduction

Multiple sclerosis (MS) is a chronic immune-mediated disease of the central nervous system characterized by substantial clinical heterogeneity in inflammatory activity, clinical course, disability accumulation, and treatment response (1, 2). In relapsing forms of MS, particularly relapsing-remitting multiple sclerosis (RRMS), this heterogeneityis directly relevant to clinical decision-making because relapse activity, radiological activity, and disability worsening vary substantially across patients and over time (3, 4). Individualized estimation of future disease activity has therefore become a major objective of contemporary MS research, with potential implications for treatment escalation, monitoring intensity, patient counseling, and clinical trial enrichment (2, 5, 6).

Traditional prognostic assessment in MS has relied on a limited set of clinical and paraclinical indicators, including prior relapse history, disability burden, disease duration, and magnetic resonance imaging (MRI) lesion activity (7). Although these variables remain clinically informative, their predictive value is modest when used in isolation for individualized risk estimation. The growing availability of electronic health records (EHRs), multicenter registries, randomized clinical trial datasets, biomarker platforms, and multimodal imaging data has led to a rapid expansion of multivariable prediction research in MS. This expansion has been accompanied by increasing use of penalized regression, Bayesian modeling, machine learning (ML), deep learning, and treatment-effect heterogeneity frameworks intended to improve individualized estimation of relapse risk, disease activity or treatment benefit (8).

However, the current evidence base remains methodologically and conceptually heterogeneous. Studies differ in population, data modality, analytical unit, endpoint definition, prediction horizon, and validation strategy. Some focus on future relapse risk, whereas others address annualized relapse rate, broader disease activity, biomarker-associated outcomes, or individualized treatment benefit. As a result, the literature is clinically relevant but not directly comparable across all included studies, and reported performance metrics cannot be interpreted without considering endpoint definition, analytical unit, and validation design (9, 10).

The aim of this systematic review was to identify and critically compare models addressing relapse-related outcomes in relapsing multiple sclerosis. Specifically, we examined differences in clinical purpose, target estimand, data source, predictor structure, modeling approach, calibration, validation strategy, and practical interpretability. We also assessed whether apparently superior predictive performance was supported by adequate validation and whether consistent predictor domains emerged across conventional prognostic and treatment-effect prediction frameworks.

## 2 Methods

This study was conducted as a systematic review in accordance with the Preferred Reporting Items for Systematic Reviews and Meta-Analyses (PRISMA 2020) guidelines (11). The objective was to identify and synthesize studies focused on prediction modeling in multiple sclerosis (MS), with emphasis on relapse-related outcomes. The review protocol was registered in the PROSPERO database (CRD42024625392).

### 2.1 Search strategy and data sources

A literature search was performed across four electronic databases: PubMed, Web of Science, IEEE Xplore, and Scopus. The search strategy was designed to identify studies applying artificial intelligence, machine learning, or traditional prediction models in MS, with a specific focus on relapse or relapse-related disease activity. The final database export was conducted on 14 January 2026 and covered English-language records published from 1 January 2010 to 14 January 2026. Search queries combined terms related to artificial intelligence and machine learning, multiple sclerosis, relapse and relapsing phenotypes, and prediction or prognostic modeling. Database-specific syntax was applied. Full search strategies are provided in Supplementary materials.

The search yielded 1,747 records (PubMed *n* = 294; Web of Science *n* = 424; IEEE Xplore *n* = 157; Scopus *n* = 872). After duplicate removal (*n* = 510), 1,237 records remained for screening. Targeted Google Scholar searches were conducted as a supplementary search using the same or closely related keyword concepts as those applied in the database searches, including terms related to multiple sclerosis, relapse, prediction or prognostic modeling, machine learning, and artificial intelligence. Google Scholar was used to identify potentially relevant records not captured by the four primary databases. Citation searching of relevant records was also used as a supplementary pathway for identifying additional studies. These supplementary approaches identified four additional studies.

### 2.2 Study selection

Study selection was performed in two stages. First, titles and abstracts (*n* = 1,237) were screened for relevance. Studies unrelated to MS, lacking prediction modeling, or not addressing relapse or relapse-related outcomes were excluded. This resulted in the exclusion of 1,114 records and the identification of 123 reports for full-text retrieval.

**Figure 1:**
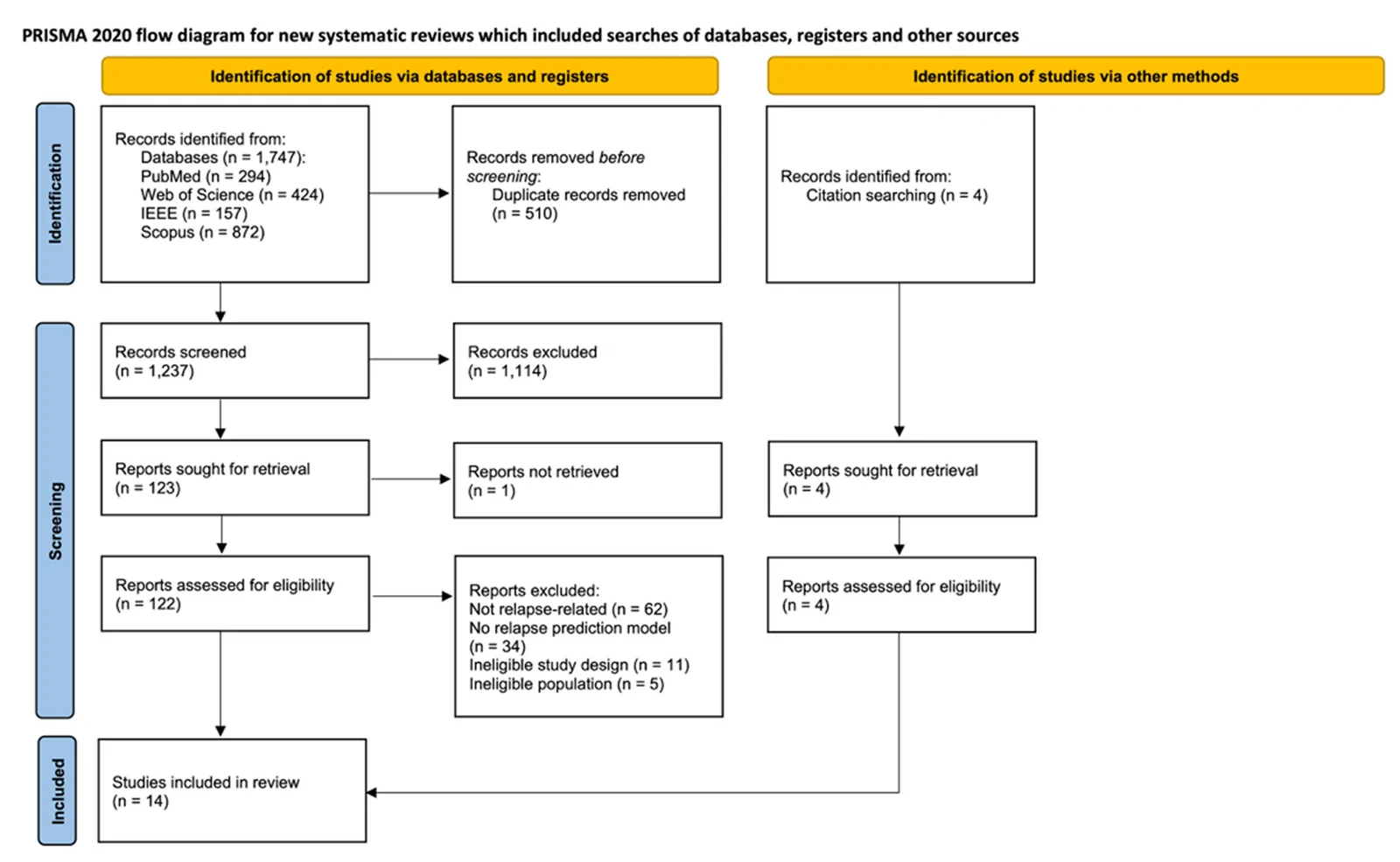
PRISMA 2020 flow diagram of the study selection process. Adapted from the PRISMA 2020 flow diagram template and populated with data from the present systematic review. Citation: Page et al. (11). License: CC BY 4.0. Source: PRISMA 2020 flow diagram (https://www.prisma-statement.org/prisma-2020-flow-diagram).

Full-text articles were assessed using predefined eligibility criteria. One report could not be retrieved, resulting in 122 reports assessed from the primary search. Four additional studies identified through citation searching were also assessed, resulting in 126 full-text evaluations. Screening and full-text assessment were performed by one reviewer and independently checked by a second reviewer, with disagreements resolved by a third reviewer. The study selection process is summarized in Figure 1.

### 2.3 Eligibility criteria

Studies were included if they involved patients with relapsing forms of MS, applied a predictive modeling framework, and included a model explicitly predicting relapse or relapse-related outcomes, regardless of whether relapse was the primary or secondary study endpoint.

Eligible populations were required to include patients with relapsing forms of MS, particularly relapsing–remitting multiple sclerosis (RRMS). Studies explicitly focusing on non-relapsing phenotypes, such as clinically isolated syndrome (CIS) without established relapsing disease or purely progressive forms without relapse activity, were excluded. In cases where the study population was described broadly as “multiple sclerosis” without explicit phenotype stratification, studies were considered eligible only if relapse-related outcomes were modeled separately and were consistent with relapsing disease activity.

In studies reporting multiple clinical outcomes, inclusion required that relapse or a relapse-related endpoint be modeled as a distinct prediction target. Studies in which relapse was only incorporated as part of a composite outcome or not independently modeled or reported were excluded, ensuring that reported performance metrics were directly attributable to relapse-related prediction.

### 2.4 Data extraction and synthesis

For each included study, data were extracted on study design, population, analytical unit, data source, prediction horizon, prediction target, modeling approach, validation strategy, and performance metrics. Where available, information on missing-data handling, feature selection, class imbalance handling, calibration reporting, external validation, and model interpretability were also extracted.

A formal prediction-model-specific risk-of-bias assessment using PROBAST or PROBAST+AI was not undertaken. The studies identified through the systematic search were substantially heterogeneous in their clinical purpose, target estimand, analytical unit, endpoint definition, and modeling framework, which limited the consistent application of a single prediction-model-specific risk-of-bias tool across the full evidence base. Instead, we performed a structured methodological appraisal focused on validation design, calibration reporting, sample size, missing-data handling, class-imbalance handling, model transparency, and clinical interpretability. This appraisal was used to support comparative interpretation rather than to assign formal risk-of-bias ratings.

Due to substantial heterogeneity in study design, endpoints, analytical units, prediction horizons, validation settings, and modeling approaches, a quantitative meta-analysis was not performed. Pooling was considered inappropriate because discrimination and performance estimates arose from non-equivalent outcomes and estimands, including conventional prognosis, treatment-effect prediction, annualized relapse-rate classification, biomarker-associated disease activity, and exploratory relapse-related tasks.

Instead, a structured narrative synthesis was conducted. For comparative interpretation, the included studies were organized into clinically defined evidence groups based on their primary clinical purpose, enabling consistent comparison across analytically similar groups. These groups included relapse prognosis, treatment-effect prediction, and exploratory relapse-related analyses.

A total of 14 studies were included in the final synthesis, including 10 identified through database searches and 4 identified through citation searching (Figure 1).

## 3 Results

### 3.1 Overview of included studies

Fourteen studies were included and stratified into three clinically defined evidence groups: five relapse-prognosis studies, four individualized treatment-effect prediction studies, and five exploratory relapse-related studies. Comparisons were made within these groups because the included studies differed substantially in estimand, endpoint definition, analytical unit, prediction horizon, and validation context. The evidence groups used for the comparative synthesis are summarized in Table 1.

**Table 1:** Evidence groups used for comparative synthesis.

Evidence group	Definition	Included studies	Role in synthesis
Core relapse-prognosis studies	Studies primarily designed to predict future relapse or relapse risk within a prognostic framework	Ahuja et al. (12); Chalkou et al. (13); Ihalapathirana et al. (14); Vakili et al. (15); Walsh et al. (16)	Primary relapse-prognosis evidence
Treatment-effect prediction studies	Studies primarily designed to estimate individualized treatment benefit, treatment response, or heterogeneous treatment effects	Chalkou et al. (17); Irmak Ön et al. (18); Jiang et al. (19); Stühler et al. (20)	Precision-medicine evidence
Exploratory relapse-related studies	Studies addressing relapse-related activity through biomarkers, imaging, environmental data, or exploratory modeling frameworks	Bejarano et al. (21); Harris et al. (22); Marinello et al. (23); Marinello et al. (24); Du et al. (25)	Contextual exploratory evidence

Among the core relapse-prognosis studies, models primarily used structured clinical or EHR-derived data and recurrent predictor domains included prior relapse activity, disease duration, disability-related measures, treatment history, and MRI variables. Predictive performance was generally moderate in the more clinically oriented models, while validation depth varied substantially across studies (12–16).

Treatment-effect prediction studies focused on individualized comparative treatment benefit rather than relapse risk alone and were based mainly on trial or registry data. These models incorporated baseline clinical characteristics and treatment-related variables, but calibration and independent validation were inconsistently reported (17–20).

Exploratory relapse-related studies extended prediction to imaging, biomarkers, environmental exposures, and challenge-based data. Although some reported comparatively high performance, these studies generally relied on more specialized data sources, smaller samples, or outcomes less directly aligned with longitudinal relapse-risk prediction (21–25).

Across clinically oriented studies, recurring predictor domains included prior relapse activity, disability-related measures, MRI activity, disease duration, and treatment history. Study architecture, predictor domains, modeling strategies, endpoints, calibration, and validation are detailed in Tables 2–4.

**Table 2:** Study architecture and clinical context of the included studies.

References	Year	Data environment	Population/analytical unit	Sample size	Prediction horizon/task structure
Ahuja et al. (12)	2021	Registry/EHR	MS with relapse-related outcome, patient	1,435 training; 186 validation	1 year
Bejarano et al. (21)	2011	Imaging cohort	MS with relapse-related outcome, patient	51 MS + 20 controls; 96 validation MS	2 years
Harris et al. (22)	2021	Biomarker trial analysis	RRMS, patient	1,244 SUNBEAM and 1,109 RADIANCE baseline pNfL samples	12–24 months
Chalkou et al. (13)	2021	Registry/EHR	RRMS, cycle	935 patients; 1,752 2-year cycles	2 years
Chalkou et al. (17)	2021	IPD from randomized trials/network meta-regression	RRMS, patient	3,590 RCT patients; additional placebo IPD for risk modeling	2 years
Ihalapathirana et al. (14)	2023	Registry/EHR	RRMS, unclear	Not clearly reported	Not clearly reported
Irmak Ön et al. (18)	2025	Randomized trial	Active RRMS, patient	843 development; 713 validation	2 years
Jiang et al. (19)	2023	Registry/EHR	RRMS, patient	2,791	Variable follow-up
Marinello et al. (23)	2024	Challenge dataset	MS with relapse-related outcome, patient	197 unique patients	Challenge-based task
Marinello et al. (24)	2024	Environmental dataset	MS, Patient-event	802 analytic sample; 814 source dataset	1-week pre-event window
Du et al. (25)	2023	Imaging cohort	RRMS, patient	25	ARR classification without longitudinal prediction horizon
Stühler et al. (20)	2020	Registry/EHR	RRMS, cycle	3,119 development/validation cycles; 314 test cycles	Treatment-course based
Vakili et al. (15)	2023	Registry/EHR	RRMS, record	492 records from 84 patients	1 year
Walsh et al. (16)	2022	Registry/EHR	PwMS, patient	2,532	1-year analysis window

ARR, annualized relapse rate; EHR, electronic health records; IPD, individual participant data; MS, multiple sclerosis; pNfL, plasma neurofilament light chain; PwMS, people with multiple sclerosis; RCT, randomized controlled trial; RMS, relapsing multiple sclerosis; RRMS, relapsing-remitting multiple sclerosis. “Not clearly reported” indicates insufficient reporting in the original article or unclear disease-specific analytic denominator.

**Table 3:** Input data and modeling strategy.

References	Data modalities	Core predictor domains	Modeling approach
Ahuja et al. (12)	Structured clinical data	Age, disease duration, imputed prior 1-year relapse history	Two-stage LASSO logistic regression
Bejarano et al. (21)	Clinical + MRI + neurophysiology	EDSS, MRI lesion load, CMCT, gray matter measures	Multiple classifiers including neural network
Harris et al. (22)	Biomarker + MRI + clinical outcomes	pNfL, MRI lesion activity, brain volume, treatment-related pNfL change	Biomarker association modeling
Chalkou et al. (13)	Structured clinical data + MRI	Age, sex, prior treatment, time since last relapse, disease duration, prior relapses, EDSS, Gd-enhancing lesions	Bayesian mixed-effects modeling
Chalkou et al. (17)	Trial clinical data	Baseline relapse-risk score, age, disability status, treatment-specific interaction structure	Two-stage risk modeling + network meta-regression
Ihalapathirana et al. (14)	Structured clinical data	Clinical, socioeconomic, physical health, mental health, and sex-stratified predictors	Boosting/tree-based ML with SHAP
Irmak Ön et al. (18)	Trial clinical data + MRI	EDSS, Gd-enhancing T1 lesion volume, relapses in last 2 years, prior MS treatments	Penalized survival modeling + tree methods
Jiang et al. (19)	Structured clinical data	Baseline clinical and treatment variables, propensity/precision-medicine scores, treatment-effect modifiers	Precision-medicine scoring framework
Marinello et al. (23)	Environmental data + static variables	Environmental descriptors and static patient variables	Logistic regression, ridge regression, random forest
Marinello et al. (24)	Structured clinical data + environmental data	Weather, pollutants, disability, treatment timing	Logistic regression + random forest
Du et al. (25)	PET/MR + radiomics	PET/MR lesion radiomics features, automated lesion segmentation features, selected imaging features	Deep-learning radiomics pipeline
Stühler et al. (20)	Structured clinical data	Age, sex, EDSS, relapse history, treatment history, therapy duration, clinical site	Hierarchical Bayesian modeling
Vakili et al. (15)	Structured clinical data	Relapse symptom categories, demographic/personal variables, clinical symptom records	Multiple supervised ML models
Walsh et al. (16)	Structured clinical data	Demographics, MS phenotype, center-level variables, DMT, MRI use, EHR-derived clinical variables	Logistic regression, penalized regression, random forest

CMCT, central motor conduction time; DMT, disease-modifying therapy; EDSS, Expanded Disability Status Scale; Gd, gadolinium; MRI, magnetic resonance imaging; ML, machine learning; PET/MR, positron emission tomography/magnetic resonance; pNfL, plasma neurofilament light chain; SHAP, Shapley additive explanations.

**Table 4:** Target endpoints, calibration reporting, and validation depth.

References	Target endpoint	Main metric(s)	Calibration assessment	External or independent validation
Ahuja et al. (12)	1-year relapse	AUC 0.707; F-score 0.307	Descriptive calibration examined; standard calibration slope/intercept reported	Held-out independent validation within the study dataset
Bejarano et al. (21)	EDSS change, disability progression, relapse	~80% accuracy for EDSS change; relapse-specific performance not separately reported	Not reported	Validation in a second MS cohort
Harris et al. (22)	ARR, MRI activity, brain volume loss, NEDA	pNfL reduction associated with improved outcomes	Not applicable/not reported as prediction-model calibration	Not reported
Chalkou et al. (13)	Relapse within 2 years	c-statistic 0.65; calibration slope 0.92	Calibration slope reported	Internal validation only
Chalkou et al. (17)	Treatment-specific probability of relapse within 2 years	Stage-1 baseline-risk model: c-score 0.60–0.62; treatment-specific benefit estimates from network meta-regression	Not reported	Not reported
Ihalapathirana et al. (14)	Relapse prediction	F1 84.55% in males; 76.11% in females; ROC-AUC 70.26%/69.05%	Not reported	Not reported
Irmak Ön et al. (18)	Time-to-relapse, MRI lesions, CDP, safety	Relapse AUC 0.68; T2 lesion AUC 0.74; CDP AUC 0.59	Calibration plots, intercept, and slope reported; relapse slope 1.06	External validation in an independent RCT cohort (FREEDOMS II)
Jiang et al. (19)	ARR, time to first relapse, CDP	Patient-specific comparative effectiveness estimates; no single discrimination metric	Not reported	Not reported
Marinello et al. (23)	Relapse prediction task	Challenge RMSE improved from 72.992 to 69.564 after adding environmental data	Not reported	Challenge test set; not external validation in an independent clinical population
Marinello et al. (24)	Imminent relapse	AUC-ROC 0.713; AUC-PR 0.639	Not reported	Not reported
Du et al. (25)	ARR classification	AUC 0.96; ACC 0.88; DSC 0.81	Not reported	Not reported
Stühler et al. (20)	Treatment-course response: relapses and CDP	Relapse predictive C-index 0.646; test-set C-index 0.608	No standard calibration metric; MSE/NLL reported	Independent test cycles/site-based validation as reported
Vakili et al. (15)	Relapse during 1-year analysis window	SVC Precision 98.837%; ACC 97.973%	Not reported	Not reported
Walsh et al. (16)	Relapse during 1-year analysis window	RF AUC 0.69 vs. logistic regression AUC 0.63; F1 up to 0.20	Not reported	Not reported

ACC, accuracy; ARR, annualized relapse rate; AUC, area under the receiver operating characteristic curve; AUC-PR, area under the precision–recall curve; CDP, confirmed disability progression; DSC, Dice similarity coefficient; EDSS, Expanded Disability Status Scale; MRI, magnetic resonance imaging; MSE, mean squared error; NEDA, no evidence of disease activity; NLL, negative log-likelihood; RCT, randomized controlled trial; RF, random forest; RMSE, root mean squared error; ROC-AUC, receiver operating characteristic area under the curve; SVC, support vector classifier. Metrics are reported as presented in the original studies and should not be interpreted as directly comparable across heterogeneous endpoints, prediction horizons, analytical units, and validation settings.

### 3.2 Cross-study patterns

The stratified synthesis showed that relapse-related prediction studies cannot be interpreted as a single homogeneous evidence base. Conventional relapse-prognosis models estimated future relapse risk using routinely available clinical variables, whereas individualized treatment-effect studies addressed patient-specific comparative benefit and therefore targeted a different estimand (12, 13, 16–20). Exploratory biomarker, imaging, environmental, and challenge-based studies added potentially relevant predictor domains but were less directly aligned with routine relapse-risk prediction and often relied on more specialized data sources (21–25).

Across clinically oriented studies, the most recurrent predictor domains were prior relapse activity, disability-related measures, disease duration, MRI activity, and treatment history. However, apparent predictive performance was closely linked to validation depth. Models based on structured clinical data and more transparent validation generally reported moderate discrimination, whereas the highest performance estimates were typically observed in smaller, high-dimensional, or less externally validated studies (12, 15, 17, 18, 20, 25).

From a methodological perspective, the highest reported performance should therefore be interpreted cautiously. For example, very high classification performance was observed in some small or high-dimensional studies, whereas clinically oriented models with more extensive validation generally showed more moderate discrimination. This contrast suggests that apparent predictive performance alone is insufficient to establish model quality, particularly when calibration is unreported and independent validation is absent. Sample size, predictor dimensionality, calibration, and validation design should therefore be considered alongside discrimination metrics when assessing the strength of the available evidence.

## 4 Discussion

This systematic review identified three clinically distinct forms of relapse-related modeling in relapsing multiple sclerosis: conventional relapse prognosis, individualized treatment-effect prediction, and exploratory relapse-related modeling. The most methodologically credible evidence was derived from structured clinical datasets and demonstrated moderate rather than high discrimination. By contrast, the largest performance estimates were generally reported in smaller or high-dimensional studies with limited calibration assessment and independent validation.

This contrast highlights an important trade-off in the current evidence base: models developed in larger clinical or trial-derived datasets tended to provide more modest but methodologically more credible performance estimates, whereas highly flexible or high-dimensional approaches sometimes produced stronger apparent performance at the cost of greater uncertainty regarding overfitting and generalizability (26). These findings indicate that the principal challenge is not simply the development of increasingly complex algorithms, but the demonstration of transportability, calibration, and clinical utility across populations and settings (27, 28).

The heterogeneity in reported model performance also reflects substantial variation in prediction targets, analytical units, relapse definitions, prediction horizons, predictor dimensionality, and validation design. These differences limit direct comparison of discrimination metrics across studies and may partly explain the wide range of reported performance (29). Accordingly, higher AUC or accuracy should not necessarily be interpreted as evidence of superior clinical performance without considering calibration, sample size, and validation in independent populations (26, 30, 31).

A critical limitation across the current literature is the reliance on relatively small, often single-center cohorts, which restricts both statistical power and generalizability. In many domains of clinical AI, small datasets are known to impair model robustness and increase the risk of unstable or biased predictions. Incomplete data capture, inconsistent acquisition protocols, and missing modalities further increase the risk of overfitting, particularly in flexible architectures such as deep neural networks. As a result, reported predictive performance frequently fails to reproduce when applied to independent or more heterogeneous patient populations, reflecting a broader generalization gap in clinical AI systems (32–35).

In this review, internal validation refers to resampling or data splitting within the development dataset, site-based validation refers to testing in a separate sample or site that remains closely related to the development setting, whereas true external validation refers to evaluation in an independent population that differs meaningfully in geography, time, clinical setting, or patient composition. This distinction is important because validation within the development data or a closely related setting provides weaker evidence of model transportability than evaluation in an independent population. Performance demonstrated through internal resampling, data splitting, or validation within closely related study populations does not establish that a model will retain its discrimination and calibration when applied across different clinical centers, healthcare systems, patient populations, or treatment patterns (29, 30). For relapse prediction in MS, this is especially relevant because patient characteristics, treatment exposure, MRI acquisition, follow-up schedules, and clinical documentation may vary substantially between settings (29, 36). Future studies should therefore prioritize geographically and temporally independent validation and explicitly evaluate both discrimination and calibration before models are considered for clinical use (30).

Beyond dataset limitations, substantial heterogeneity exists in study design, outcome definitions, and model evaluation strategies. Relapse-related outcomes were defined across different time horizons and analytical levels, including patient-, cycle-, record-, and event-level analyses. In the context of MS, even well-established biomarkers such as MRI measures show variable predictive value and require careful interpretation, illustrating the complexity of linking imaging findings to clinical outcomes. Furthermore, many studies emphasize discrimination performance, such as AUC or accuracy, while neglecting calibration and clinical utility, which are essential for real-world applicability of predictive models (37).

Given these methodological and data-related inconsistencies, a formal meta-analysis was not considered appropriate. The structured narrative synthesis instead enabled comparison within clinically defined evidence groups while preserving distinctions in estimand, endpoint definition, analytical unit, and validation context. The principal contribution of this review is therefore not a pooled estimate of predictive performance, but a clinically oriented framework distinguishing conventional relapse prognosis, individualized treatment-effect prediction, and exploratory relapse-related modeling.

The limitations identified in this review highlight the need for larger, more standardized, and systematically curated datasets. National and multinational multicenter registries may help address these challenges by providing longitudinal and multimodal data across diverse patient populations. The Czech national registry ReMuS is one example of such infrastructure, combining clinical, demographic, and treatment data from multiple sclerosis centers nationwide (38, 39). Experience from large MS registries and collaborative data networks further illustrates both the potential and the methodological requirements of such infrastructures. Initiatives linking national registries and international MS datasets have emphasized harmonization of core data elements, standardized definitions, and common data models as prerequisites for robust cross-registry analyses and large-scale real-world evidence generation. Such collaborative infrastructures may also provide an important basis for developing and externally validating prediction models across heterogeneous healthcare settings (40, 41). However, the value of registry-based modeling will depend on harmonized outcome definitions, transparent missing-data handling, consistent documentation of treatment exposure, and validation across independent clinical settings. Comparable large-scale registry and trial-derived datasets may also support rigorous validation of prediction models, including deep learning approaches, across clinically heterogeneous populations (42).

Clinical implementation will require more than adequate predictive performance (26). Relapse-prediction models would need to provide risk estimates that correspond to clinically actionable decisions, such as adjustment of monitoring intensity, additional clinical or imaging assessment, or consideration of treatment modification (30, 43). This requires predefined decision thresholds, assessment of calibration and clinical utility, and integration with routinely available clinical information rather than reliance on highly specialized predictors that may not be consistently available across centers. Prospective evaluation within clinical workflows will ultimately be necessary to determine whether model-assisted prediction improves decision-making or patient outcomes compared with standard clinical assessment alone (26, 44).

From a digital health perspective, clinically validated relapse-prediction models could be integrated into EHR- or registry-based clinical decision-support workflows, where routinely collected clinical information is used to generate patient-level risk estimates at clinically relevant time points. Rather than replacing clinical judgement, these estimates could support clinician assessment by identifying patients who may warrant closer monitoring, additional clinical or imaging assessment, or reconsideration of treatment strategy. Individualized treatment-effect models would serve a distinct role by estimating the expected relative benefit of alternative treatment options rather than relapse risk alone. Effective digital integration would therefore require clear presentation of model outputs, explicit linkage between predicted risk or treatment benefit and the intended clinical action, compatibility with local clinical workflows, and continued clinician oversight (44, 45). Following deployment, model performance should be monitored over time and across clinical settings because changes in patient populations, treatment patterns, data capture, and local workflows may affect model validity and transportability (45, 46).

To ensure that future predictive modeling studies achieve clinical relevance, greater emphasis should also be placed on methodological transparency and standardization. Reporting frameworks such as TRIPOD provide structured guidance for the transparent reporting of prediction model studies, while extensions such as TRIPOD+AI adapt these principles to machine learning-based approaches. Similarly, tools such as PROBAST support systematic assessment of risk of bias and applicability in prediction models. Their consistent application would improve reproducibility, facilitate cross-study comparisons, and support the translation of predictive models into clinical practice (26, 47).

Overall, future progress is likely to depend less on algorithmic complexity alone than on rigorous model development, transparent reporting, and clinically meaningful evaluation (26). AI-based approaches, including deep learning, may be particularly valuable for integrating longitudinal and multimodal information from clinical records, imaging, biomarkers, and potentially environmental data, provided that sufficiently large and representative datasets are available (48, 49). However, increasing model complexity should be justified by demonstrable improvement over simpler clinical models and should not come at the expense of interpretability, calibration, or transportability (43, 46). Future research should prioritize multicentre development and external validation, standardized relapse definitions and prediction horizons, prospective evaluation in clinical workflows, and assessment of whether model-guided decisions provide incremental clinical benefit beyond established predictors (30, 43, 44).

### 4.1 Limitations of the review

This review has several limitations. First, the included studies were highly heterogeneous in clinical purpose, target endpoint, prediction horizon, analytical unit, data source, and validation strategy, which precluded quantitative pooling and limited direct comparison of reported performance metrics.

Second, the review combined conventional relapse-prognosis models, individualized treatment-effect models, and exploratory relapse-related analyses. Although these studies were stratified into clinically defined evidence groups, their estimands were not fully equivalent and the resulting synthesis should therefore be interpreted cautiously.

Third, screening and full-text assessment were performed by one reviewer and independently checked by a second reviewer rather than completed independently in duplicate throughout the entire selection process, which may have increased the risk of selection error.

Fourth, no formal risk-of-bias assessment using PROBAST or PROBAST+AI was undertaken because of the methodological heterogeneity and differing analytical purposes of the included evidence groups. The methodological appraisal should therefore be interpreted as a structured comparative assessment rather than as a formal risk-of-bias classification. In addition, several studies incompletely reported missing-data handling, class imbalance, calibration, and external validation, restricting the depth of comparative appraisal.

Finally, supplementary citation searching and targeted Google Scholar searches may have introduced some selection bias despite the use of predefined eligibility criteria.

## 5 Conclusion

Relapse-related prediction in relapsing multiple sclerosis is methodologically feasible, but current models are not yet supported by sufficiently consistent evidence for routine clinical implementation. The most credible studies used structured clinical predictors and transparent validation but generally achieved only moderate discrimination. In contrast, very high-performance estimates were commonly reported in smaller exploratory studies without robust external validation. The major strengths, limitations, and potential clinical applicability of the included studies are summarized in Table 5. Future progress will depend on harmonized outcome definitions, adequate calibration, independent validation, and explicit separation of conventional prognostic models from individualized treatment-effect models. The principal challenge is therefore shifting from proof of concept toward transportable and clinically useful prediction.

**Table 5:** Critical appraisal of the included studies: major strengths, limitations, and potential clinical applicability.

References	Major strengths	Major limitations	Potential clinical applicability
Ahuja et al. (12)	Large EHR dataset; interpretable LASSO model; calibration and held-out independent validation reported	Moderate discrimination (AUC 0.707; F-score 0.307); broader external validation not reported	Potentially applicable to 1-year relapse-risk estimation using routinely available structured clinical data; validation across additional settings is needed
Bejarano et al. (21)	Multimodal clinical, MRI, and neurophysiological predictors; validation in a second MS cohort	Small development sample; calibration not reported; relapse-specific predictive performance not reported separately	May support multimodal short-term disease-course assessment, but relapse-specific clinical utility remains uncertain
Harris et al. (22)	Large trial-derived biomarker dataset; clinically relevant integration of pNfL, MRI, and clinical outcomes	Prediction-model calibration not applicable/not reported; no independent prediction-model validation reported	Potential relevance for biomarker-supported monitoring of disease activity rather than direct, validated relapse-risk prediction
Chalkou et al. (13)	Clinically interpretable clinical/MRI predictors; calibration slope reported; substantial registry/EHR sample	Moderate discrimination (c-statistic 0.65); internal validation only	Potentially applicable to 2-year relapse-risk estimation with routine clinical and MRI variables; external validation is required before broader use
Chalkou et al. (17)	Large randomized-trial IPD dataset; individualized treatment-effect framework	Different estimand from conventional prognosis; baseline-risk discrimination was moderate; calibration and independent validation not reported	Potentially relevant to individualized treatment selection by estimating comparative benefit; requires independent validation before routine clinical use
Ihalapathirana et al. (14)	Explainable ML approach using SHAP; incorporates clinical, socioeconomic, physical-health, mental-health, and sex-stratified predictors	Sample size, analytical unit, and prediction horizon not clearly reported; calibration and independent validation not reported	Potential for interpretable relapse-risk estimation and subgroup exploration, but incomplete reporting limits assessment of clinical implementation
Irmak Ön et al. (18)	Trial-derived development and external validation cohorts; calibration reported; combines clinical and MRI predictors	Relapse discrimination remained moderate (AUC 0.68); applicability beyond trial populations requires further evaluation	Relatively strong translational potential for time-to-relapse and treatment-response assessment because calibration and external validation were reported; real-world validation remains desirable
Jiang et al. (19)	Large registry dataset; patient-specific comparative-effectiveness framework using routine clinical and treatment variables	Different estimand from conventional relapse prognosis; no single discrimination metric; calibration and independent validation not reported	Potentially useful for individualized comparative treatment-effect estimation and treatment selection; prospective or independent validation is needed
Marinello et al. (23)	Adds environmental information to relapse-related prediction; evaluated on a challenge test set	Relatively small cohort; calibration not reported; challenge test set is not conventional external validation	Exploratory value for incorporating environmental exposures into relapse-related models; direct routine clinical applicability is currently limited
Marinello et al. (24)	Integrates clinical and environmental predictors for short-term imminent-relapse assessment; moderate reported discrimination	Calibration and independent validation not reported	Potential for short-term risk alerts combining environmental exposure with clinical information; external and prospective validation are required
Du et al. (25)	Multimodal PET/MR radiomics with automated lesion features; high reported classification performance	Very small sample (*n* = 25); calibration and independent validation not reported; ARR classification rather than longitudinal relapse prediction	Proof-of-concept for imaging-based ARR classification; current sample size and validation depth substantially limit clinical use
Stühler et al. (20)	Large registry/EHR dataset; hierarchical Bayesian modeling; independent test cycles/site-level validation reported	Standard calibration metrics not reported; test-set discrimination was moderate; treatment-response estimand differs from conventional prognosis	Potentially relevant to personalized treatment-response prediction using routine clinical variables; further calibration and external validation would strengthen implementation
Vakili et al. (15)	Uses structured clinical data and reports very high precision and accuracy across supervised ML models	Small patient sample relative to the number of records; calibration and independent validation not reported; potential overfitting concern	Promising proof-of-concept for 1-year relapse prediction, but patient-level external validation and calibration are needed before clinical use
Walsh et al. (16)	Large EHR cohort; direct comparison of logistic, penalized-regression, and random-forest approaches using routine clinical variables	Calibration and independent validation not reported; discrimination was moderate and F1 scores were low	Useful benchmark for EHR-based 1-year relapse-risk prediction; prospective external validation and calibration are needed before deployment.

Comments summarize the structured comparative appraisal described in the Methods and should not be interpreted as formal risk-of-bias ratings. ARR, annualized relapse rate; AUC, area under the receiver operating characteristic curve; EHR, electronic health records; IPD, individual participant data; MRI, magnetic resonance imaging; pNfL, plasma neurofilament light chain; SHAP, Shapley additive explanations.
