# Supplementary material for: Relapse prediction and individualized treatment-effect modeling in relapsing multiple sclerosis: a systematic review
Source: Front Public Health. 2026 Sep 4;14:1940704. doi: 10.3389/fpubh.2026.1940704 (PMC13587037; doi:10.3389/fpubh.2026.1940704)
Supplement: Supplementary file 1 [file Table_1.docx]

| Search query | |
| --- | --- |
| PubMed | (("artificial intelligence" OR "machine learning" OR "deep learning" OR "neural networks" OR "AI" OR "ML" OR "Decision Tree" OR "Random Forest" OR "Machine Intelligence" OR "Support Vector Machine") AND ("Multiple sclerosis" OR "MS") AND ("Relapse" OR "Relapsing-remitting" OR "RRMS" OR "Relapsing-remitting multiple sclerosis (RRMS)" OR "Relapsing-remitting multiple sclerosis" OR "Multiple sclerosis relapse" OR "Relapsing multiple sclerosis" OR "exacerbation" OR "Flare-up") AND ("Prediction" OR "Predictive" OR "Predict" OR "Prediction model" OR "Predictive model" OR "Probability" OR "Prognosis" OR "Prognostic" OR "Predicted" OR "Risk" OR "Risk Assessment" OR "Predictive value of tests" OR "Precision")) |
| Web of Science | TS=("artificial intelligence" OR "machine learning" OR "deep learning" OR "neural networks" OR "AI" OR "ML" OR "Decision Tree" OR "Random Forest" OR "Machine intelligence" OR "Support Vector Machine") AND TS=("Multiple sclerosis" OR "MS") AND TS=("Relapse" OR "Relapsing-remitting" OR "RRMS" OR "Relapsing-remitting multiple sclerosis (RRMS)" OR "Relapsing-remitting multiple sclerosis" OR "Multiple sclerosis relapse" OR "Relapsing multiple sclerosis" OR "exacerbation" OR "attack" OR "flare-up") AND TS=("Prediction" OR "Predictive" OR "Predict" OR "Prediction model" OR "Predictive model" OR "Probability" OR "Prognosis" OR "Prognostic" OR "Predicted" OR "Risk" OR "Risk Assessment" OR "predictive value of tests" OR "precision") AND LA=English |
| IEEE | ("artificial intelligence" OR "machine learning" OR "deep learning" OR "neural networks" OR "AI" OR "ML" OR "Decision Tree" OR "Random Forest" OR "Machine Intelligence" OR "Support Vector Machine") AND ("Multiple sclerosis" OR "MS") AND ("Relapse" OR "Relapsing-remitting" OR "RRMS" OR "Relapsing-remitting multiple sclerosis (RRMS)" OR "Relapsing-remitting multiple sclerosis" OR "Multiple sclerosis relapse" OR "Relapsing multiple sclerosis" OR "exacerbation" OR "attack" OR "flare-up") AND ("Prediction" OR "Predictive" OR "Predict" OR "Prediction model" OR "Predictive model" OR "Probability" OR "Prognosis" OR "Prognostic" OR "Predicted" OR "Risk" OR "Risk Assessment" OR "Predictive value of tests" OR "Precision") |
| Scopus | TITLE-ABS-KEY ( ( "artificial intelligence" OR "machine learning" OR "deep learning" OR "neural networks" OR "AI" OR "ML" OR "Decision Tree" OR "Random Forest" OR "Machine intelligence" OR "Support Vector Machine" ) AND ( "Multiple sclerosis" OR "MS" ) AND ( "Relapse" OR "Relapsing-remitting" OR "RRMS" OR "Relapsing-remitting multiple sclerosis (RRMS)" OR "Relapsing-remitting multiple sclerosis" OR "Multiple sclerosis relapse" OR "Relapsing multiple sclerosis" OR "exacerbation" OR "attack" OR "flare-up" ) AND ( "Prediction" OR "Predictive" OR "Predict" OR "Prediction model" OR "Predictive model" OR "Probability" OR "Prognosis" OR "Prognostic" OR "Predicted" OR "Risk" OR "Risk Assessment" OR "predictive value of tests" OR "precision" ) ) AND PUBYEAR > 2009 AND PUBYEAR < 2025 AND ( LIMIT-TO ( LANGUAGE , "English" ) ) |
